# Adequate housing as a social determinant of the health of international migrants and locals in Chile between 2013 and 2022

**DOI:** 10.1186/s12889-024-19491-w

**Published:** 2024-07-29

**Authors:** Alice Blukacz, Marcela Oyarte, Báltica Cabieses

**Affiliations:** 1 Centro de Salud Global Intercultural, Instituto de Ciencias e Innovación en Medicina, Facultad de Medicina Clínica Alemana Universidad del Desarrollo y Facultad de Psicología, Universidad del Desarrollo, Santiago, Chile; 2 Unidad de estudios, Instituto de Salud Pública (ISP), Santiago, Chile; 3 Department of Health Sciences, University of York, York, UK

**Keywords:** Housing, Social determinants of health, International migration, Health inequities

## Abstract

**Background:**

Adequate housing is a fundamental right and a social determinant of health. It also represents a historically contentious topic in Latin America. Migratory flows to Chile have become increasingly precarious in the past few years, limiting opportunities for adequate housing, with potential repercussions on the health of international migrants and the general population. This study aims to analyse adequate housing as a social determinant of health among international migrants and locals between 2013 and 2022 in Chile.

**Methods:**

Observational cross-sectional study based on repeated versions of the nationally representative Socioeconomic Characterization Survey in Chile. Adequate housing indicators adapted from the United Nations Housing Rights Programme guidelines were analyzed with relation to individual health, distinguishing between the local and international migrant populations. Logistic regression models were fitted for housing indicators with migration as the main independent variable and for short-term and long-term healthcare needs in locals and immigrants with housing as the main dependent variables. Models were adjusted for sociodemographic variables and considered the complex sample design.

**Results:**

Descriptive findings indicated higher availability of services and infrastructure among international migrants, and a disadvantage for habitability, location, and affordability by quintiles compared to locals. Logistic regression models, adjusting for demographic variables, revealed significant associations between migration status and overcrowding (OR 6.14, 2022), poor housing materiality (OR 5.65, 2022) and proximity to healthcare centres (OR 1.4, 2022) compared to locals. Experiencing hazardous situations consistently predicted short-term healthcare needs in both migrants (OR = 1.4, 2022) and locals (OR = 2.8, 2022). Overcrowding predicted both long and short-term healthcare needs among locals across the years and long term needs among migrants in 2013 and 2015.

**Conclusions:**

We found significant inequities in adequate housing between migrant populations and locals in Chile, and some inequities among both populations based on structural socioeconomic deprivation. Experiencing hazardous situations emerged as a social determinant of health among international migrants in 2022, potentially suggesting growing challenges related to social exclusion in urban areas. However, limitations such as exclusion criteria of the survey and sample sizes for data on the migrant population potentially suggest that housing challenges and their impact on health are underestimated.

## Background

Housing is widely recognized as a social determinant of health. Beyond the dichotomy of the homeless versus the housed [1], inadequate housing can have negative repercussions on the health of the populations experiencing it [2]. The 1966 International Covenant on Economic, Social and Cultural Rights established the right to “adequate housing” as part of the right to an adequate standard of living [3]. More specifically, the Committee on Economic, Social and Cultural Rights General Comment No. 4: The Right to Adequate Housing of 1991 defines the right to adequate housing as the right to more than a roof over one’s head, adding that the concept of adequacy serves to guarantee the right to live somewhere in security, peace, and dignity [4]. In that sense, the Committee describes the right to adequate housing as encompassing the dimensions of legal security of tenure, availability of services, materials, facilities and infrastructure, affordability, habitability, accessibility, location, and cultural adequacy.

Concerning health, the existing evidence at the global level shows that aspects of inadequate housing such as unaffordability, housing instability, low-quality construction, indoor pollution, limited water supply, inadequate waste disposal system, or overcrowding, have an impact on physical and mental health, which can translate into short-term medical needs, such as needing care as a result of a burn, a fall, exposure to chemicals, or infections, as well as longer-term and chronic conditions, such as obesity, hypertension, diabetes, cardiovascular diseases, skin conditions, asthma, depression, among others [2, 5–11].

International migration is recognized as a social determinant of health, as it can influence the health of people who migrate, those left behind and the receiving communities [12]. More specifically, international migrants can experience exacerbated existing inequalities, leading to negative health outcomes [13]. The global literature shows that housing affects the physical and mental health of international migrants through similar pathways as local populations, however, the causes of inadequate housing are linked to factors specific to migratory processes, such as visa status, informal or underpaid employment, language barriers, racism and discrimination, and limited access to mechanisms to report abuse from landlords [14–16].

Inadequate housing has been a historically contentious topic in Latin America, as the region has seen rapid urbanization processes marked by deep social inequalities as a result of mass rural-to-urban migrations [17]. In Chile, processes of internal migration from rural areas to the capital city of Santiago grew during the 1950s, exceeding the capacity of the state to regulate urbanization, ensure adequate housing to the new urban population, and mitigate housing inequities, despite the creation of the Ministry of Housing and Urbanism in 1965 [18]. This led to the establishment of informal settlements and although they have been progressively formalized, new ones keep emerging to this day [19]. Furthermore, the military dictatorship starting in 1973 privatized many State-led basic services during the 1980s, and the management of urban planning and housing was no exception, leading to the city’s uncontrolled expansion and the deepening of territorial and housing inequities [20, 21]. The popular uprisings commonly called “estallido social” or “social outburst”, that took place around Chile starting in October 2019 and culminated with the new Constitutional process, included in its grievances the right to “decent housing” (“vivienda digna”) [22, 23]. These grievances remain largely unaddressed, and the COVID-19 pandemic came to dramatically highlight persisting housing inequities, where more socially vulnerable segments of society faced increased risks of infection mainly due to overcrowding [24].

Chile is home to 19 million people, 1.7 million of whom are international migrants [25]. The country has emerged as an important destination for regional immigrants from Latin America and according to the website of *Servicio Nacional de Migraciones*, the main countries of origin of migrants are Venezuela, Peru, Colombia, Haiti, and Bolivia for 2022. In the past ten years, furthermore, immigration patterns have changed. Immigrants from Venezuela now represents the largest community in the country, replacing Peru as the main country of origin and Haiti emerged as a new country of emigration to Chile [26]. However, immigration processes to Chile have become increasingly challenging for Venezuelan nationals, as additional visa requirements targeting them were established in 2018 [27, 28]. This led to an increase in irregular migration with little to no opportunity to obtain a residence permit once in Chile, and this phenomenon was exacerbated by border closures during the COVID-19 pandemic [29–31]. The precariousness of migratory trajectories, in turn, strongly limited the opportunities of an increasing number of international migrants to work in the formal labour market, earn a decent and steady income, and access adequate housing. For instance, this led to international migrants increasingly living in informal settlements or highly precarious and overcrowded housing units [32–34].

Considering the Chilean context surrounding housing and the recent evolution of migratory flows to Chile, this study aimed at analysing adequate housing as a social determinant of health among international migrants and locals between 2013 and 2022.

## Materials and methods

### Study design

An observational cross-sectional study was conducted based on repeated versions of the anonymous and nationally representative National Socioeconomic Characterization Survey (*Encuesta de Caracterización Socioeconómica Nacional*, CASEN survey thereafter). Adequate housing indicators were analyzed in relation to individual health status, distinguishing between the local and international migrant populations.

### Materials

The 2013, 2015, 2017, 2020 and 2022 versions of the CASEN survey were used. The CASEN survey is used as a diagnostic, evaluation, and targeting tool as it seeks to identify gaps and information on the socioeconomic conditions of households in Chile, especially among priority groups as defined by social policies at the national level. CASEN has been collecting demographic and socioeconomic data in Chile since the 1980s, every 2 to 3 years; it is well-accepted and consistently shows high percentages of acceptability from participants from all over the country. It is worth noting that as a result of the COVID-19 pandemic, the 2020 version of the survey was conducted in a mixed sequential mode with three phases (face-to-face pre-contact, telephone application of the questionnaire and face-to-face data recovery in specific cases), its content was reduced, and questions specific to the context of the pandemic were included.

The data are open access, collected through structured interviews with eligible informants (heads of household or a household member over 18 years old), through a probabilistic, stratified, and multistage sampling, covering topics such as household composition, education, work, income, health, identity, and housing, and being representative at national, regional and area (urban-rural) levels.

The survey is representative of the population. In 2013, the sample size for the migrant population was 3555 (representative of 354,581 people) and 212,346 for the locals (representative of 16,689,377 people), in 2015, it was 4851 for international migrants (representative of 465,319 people) and 260,754 for the locals (representative of 16,970,061 people), in 2017 it was 6811 for international migrants (representative of 777,407 people) and 207,603 locals (representative of 16,843,471), in 2020 it was 8857 international migrants (representative of 1,191,601 people) and 173,462 locals (representative of 17,972,203 people), and finally, the sample size in 2022 was 11,894 for international migrants (representative of 1,736,691 people) and 188,785 locals (representative of 17,937,742).

### Variables and indicators

International migration: An international migrant is defined by the International Organization for Migration as “any person who is moving or has moved across an international border (…) away from his/her habitual place of residence” regardless of their migratory status [35]. Following the criteria set by the CASEN survey, anyone born outside Chile was considered an international migrant and anyone born in Chile was considered a local. We use the terms international migrants in Chile and immigrants as synonymous.

Health status: Being under treatment for any pathology during the year prior to the survey (yes/no) was used as proxy for chronic or long-term healthcare needs and having had any illness or accident in the 3 months prior to the survey (yes/no) was used as proxy for short-term healthcare needs.

Adequate housing: Based on the adequate housing indicators put forward by the United Nations Housing Rights Programme (UNHRP) 2003 report [36] and on the dimensions of adequate housing described by the Committee on Economic, Social and Cultural Rights General Comment No. 4: The Right to Adequate Housing of 1991, a series of housing variables were generated and adapted according to the variables available in the different versions of the CASEN survey. As outlined in the UNHRP report, the indicators were proposed as guidance to aid the monitoring of housing rights. As some are overlapping and others may not be measurable through a survey such as CASEN, the report recommended the indicators be adapted according to data availability and purpose of the analysis. The variables used in the present analysis were constructed by the authors according to such criteria. The survey has suffered some minor modifications throughout the years, with some questions altered, removed, or added. Considering that the 2020 version of the survey was abbreviated, data is missing for that year. However, the decision was made to include it in the present analysis. Please see Table 1 for further detail.

**Table 1:** Adequate housing indicators

United Nations Housing rights programme indicators	Indicators adjusted to the individual level	Questions from CASEN used to build indicators	Years with available data
**Availability of services**,** materials**,** facilities**,** and infrastructure**			
Number:1000 households with sanitation facilities	1000 people living in housing with sanitation facilities	Does the dwelling where you live have an excreta disposal system? (1. Yes, with toilet connected to sewer; 2. Yes, with toilet connected to septic tank; 3. Yes, with sanitary latrine connected to cesspool; 4. Yes, with box over cesspool; 5. Yes, with box over ditch or canal; 6. Yes, with box connected to another system; 7. Yes, chemical toilet onsite; 8. No system available)	2013–2015–2017–2020–2022
Number:1000 households with electricity	1000 people living in housing with electricity	Does the dwelling where you live have electricity? (1. Yes, from the public grid with its own meter; 2. Yes, from the public grid with a shared meter; 3. Yes, from the public grid without a meter; 4. Yes, from your own or a community generator; 5. Yes, from solar panel; 6. Yes, from another source; 7. No electric power available)	2013–2015–2017–2022
Number: 1000 households with potable water	1000 people living in housing with public water supply	Where does the dwelling’s water come from? (Public network with own meter; 2. Public network with shared meter; 3. Public network without meter; 4. Well or waterwheel; 5. River, spring, lake, or estuary; 6. Water truck; 7. Another source)	2013–2015–2017–2020–2022
**Habitability**			
Average number of persons/room	Average number of persons/ bedroom (number of people in the household excluding live-in domestic help/number of rooms exclusively dedicated to sleeping)	Number of people in the household excluding live-in domestic help; How many rooms of each type does the dwelling have (one household per dwelling); How many rooms of each type does your household occupy in this dwelling (when more than one household per dwelling)?	2013–2015–2017–2020–2022
Number: 1000 households with more than 2 persons per room	1000 people living in housing with more than 2 people per bedroom	Number of people in the household excluding live-in domestic help; How many rooms of each type does the dwelling have (one household per dwelling); How many rooms of each type does your household occupy in this dwelling (when more than one household per dwelling)?	2013–2015–2017–2020–2022
Number: 1000 households living in temporary/ dilapidated structures	1000 people living in housing with irrecoverable materiality index	Materiality index included in the survey (1. Acceptable; 2.Recoverable; 3.Irrecoverable)	2013–2015–2017–2022
**Location**			
Number:1000 households residing near a hazardous site	1000 people who have experienced or witnessed alcohol or drug use, drug trafficking, fights or threats on public streets and/or shootings or gunshots at least a few times in the month	In the last month, have you or anyone in your household experienced or witnessed any of the following situations…: b) People using drugs or alcohol on public roads. c) People dealing drugs on public roads. d) People fighting or threatening each other on public roads. e) Shootings or gunshots.	2013–2015–2017–2022
Number:1000 persons with access to public transportation	Every 1000 persons living in housing within 8 blocks or 1 km of public transportation	Is your home within 8 blocks or 1 km of a public transportation service (bus stop, station)? (1. Yes; 2. No; 3. Don’t know)	2015–2017–2022
Average distance from home to nearest hospital	Every 1000 persons living in housing within 20 blocks or 2.5 km of a healthcare center	Is your home within 20 blocks or 2.5 km of a healthcare center (primary care or higher)? (1. Yes; 2. No; 3. Don’t know)	2015–2017–2022
Average distance from home to nearest child care centre - Average distance from home to nearest school	Every 1000 persons living in housing within 20 blocks or 2.5 km of an educational institution	Is your home less than 20 blocks or 2.5 km from an educational center (school or kindergarten)? (1. Yes; 2. No; 3. Don’t know)	2015–2017–2022
**Affordability**			
Median household monthly housing payment/ Median household monthly income (to be calculated separately according to income distribution by quintile)	Median “Home Value Approximation” Qi / Median Total Household Income Qi (Qi being the quintiles of autonomous household income from 1 to 5, with 1 being the poorest and 5 the richest).	How much dividend do you (or should you) pay (when housing is owned); How much rent do you (or should you) pay (renter); How much rent is paid in this sector for housing similar to yours (other forms of housing tenure); Total corrected household income	2013–2015–2017–2022

Additionally, sex (male/female), age (continuous variable) and healthcare provision (public/private/none/other including military healthcare system) were included as covariates.

#### Data analysis

Adequate housing indicators were calculated for the migrant and local populations separately. Similarly, adequate housing indicators were calculated for both populations stratifying by health status, including confidence intervals due to the complex nature of the sample, at a 95% confidence level.

Subsequently, explanatory logistic regression models were fitted for each of the variables corresponding to the housing indicators (dichotomised) considering immigration as the main independent variable and sex, age, urban/rural area, and household income quintile as covariates. Additionally, logistic regression models were fitted for locals and migrants separately considering short-term health need as the dependent variable and each of the housing variables (fitted in separate models) as the independent variable and sex, age, geographic area, household income quintile and health care provision as covariates. The same process was carried out for long-term health need. The corresponding goodness-of-fit for the sample design was analysed for all models.

The data were pre-processing and analysed using STATA 17 and R software (version 4.3.2), considering the complex nature of the sample (expansion factors, strata and clusters), linearized Taylor variance estimation, strata with single sampling units treated as units of certainty and a significance of 0.05. All analyses were performed at the individual level.

### Data availability and ethics

CASEN survey datasets are free of access through the following link: https://observatorio.ministeriodesarrollosocial.gob.cl. This study was part of Fondecyt Regular 1,201,461, a project funded by the Chilean government and approved by the Ethics Committee of the Faculty of Medicine of The Universidad del Desarrollo and the Ethics Committee of the Servicio de Salud Metropolitano Sur Oriente. The study complies with ethical guidelines and regulations established by the Declaration of Helsinki.

## Results

The descriptive results show that on average the international migrant population presented better indicators on the “availability of services, materials, facilities, and infrastructure” dimension, except for living in housing with sanitation facilities in 2015 and living in housing with electricity in 2022. Among people living in housing with sanitation facilities, electricity and public water supply, there could be a significant favouring international migrants compared to locals in that area (Table 2).

However, regarding the “habitability” dimension, some of the highest significant differences between locals and international migrants were observed concerning the number of people per bedroom. Specifically, in 2022, 246.71 out of every 1000 international migrants (95%CI: 219.36–276.26) lived in housing with more than two people per bedroom vs. 64.34 out of every 1000 locals (95%CI: 60.70–68.18) (Table 2).

With regards to “location”, the migrant population showed significant higher rates of experiencing hazardous situations compared to locals from 2015 onwards. For instance, in 2022, 761.75 out of 1000 migrants had witnessed alcohol and drug use, drug trafficking, fights and shootings on public roads (95%CI: 738.09–783.90), compared to 695.27 out of 1000 locals in the same situation (95%CI: 689.62–700.85). Concerning proximity to healthcare centres, public transport and educational centres, the situation was reversed, and international migrants display an advantage, showing a higher relative number of migrants close to these than the local population, with the greatest difference being proximity to healthcare centres (Table 2).

In the case of “affordability”, differences were observed between locals and international migrants; however, gaps between the two populations narrowed as the household’s autonomous income quintile grew. For instance, in 2022 the ratio of median “Home Value Approximation” to median total household income was 0.902 for international migrants versus 0.427 for locals for the poorest quintile (Q1, difference: 0.475) and 0.293 for international migrants versus 0.222 for locals for the richest quintile (Q5, difference: 0.071) (Table 2).

**Table 2:** Housing indicators among the migrant and local populations. Chile 2013–2022

Indicator	Period	Locals		International migrants	
**Availability of services**,** materials**,** facilities**,** and infrastructure**		*Indicator*	*CI95%*	*Indicator*	*CI95%*
1000 people living in housing with sanitation facilities	2013	**994.99**	(993.96–995.84)	996.29	(988.71–998.78)
	2015	995.58	(994.81–996.24)	**995.31**	(986.70–998.35)
	2017	**994.72**	(993.96–995.38)	995.99	(991.96–998.01)
	2020	**984.83**	(983.42–986.12)	986.48	(977.26–991.99)
	2022	**987.98**	(986.71–989.14)	990.35	(986.82–992.94)
1000 people living in housing with electricity	2013	**996.05**	(995.28–996.69)	996.75	(992.78–998.54)
	2015	**997.93**	(997.51–998.28)	998.57	(996.08–999.48)
	2017	**996.91**	(996.30–997.42)	997.51	(994.58–998.86)
	2022	997.17	(996.61–997.63)	**995.46**	(992.19–997.37)
1000 people living in housing with public water supply	2013	**948.72**	(945.15–952.06)	981.82	(973.87–987.38)
	2015	**952.33**	(948.38–956.00)	985.61	(976.31–991.29)
	2017	**935.44**	(930.87–939.73)	983.56	(977.97–987.76)
	2020	**936.39**	(931.97–940.54)	973.11	(961.34–981.36)
	2022	**936.18**	(933.11–939.11)	957.92	(944.85–968.00)
**Habitability**		*Indicator*	*CI95%*	*Indicator*	*CI95%*
Average number of persons/ bedroom	2013	1.59	(1.572–1.604)	**1.93**	(1.691–2.166)
	2015	1.50	(1.490–1.513)	**1.95**	(1.839–2.070)
	2017	1.45	(1.435–1.460)	**1.98**	(1.899–2,065)
	2020	1.38	(1.373–1.394)	**1.78**	(1.692–1.861)
	2022	1.32	(1.313–1.329)	**1.86**	(1.799–1.913)
1000 people living in housing with more than 2 people per bedroom	2013	146.06	(139.32–153.07)	**256.28**	(193.57–330.96)
	2015	119.81	(114.92–124.89)	**261.76**	(221.76–306.15)
	2017	103.72	(98.85–108.80)	**276.64**	(245.67–309.91)
	2020	74.82	(70.51–79.37)	**197.73**	(173.05–224.98)
	2022	64.34	(60.70–68.18)	**246.71**	(219.36–276.26)
1000 people living in housing with irrecoverable materiality index	2013	1.41	(0.80–2.47)	**1.68**	(0.99–2.85)
	2015	1.34	(1.06–1.71)	**2.00**	(0.83–4.84)
	2017	1.65	(1.33–2.06)	**2.78**	(1.62–4.77)
	2022	2.19	(1.83–2.63)	**7.21**	(4.77–10.89)
**Location**		*Indicator*	*CI95%*	*Indicator*	*CI95%*
1000 people who have experienced or witnessed alcohol or drug use, drug trafficking, fights or threats on public streets and/or shootings or gunshots at least a few times in the month	2013	703.48	(694.77–712.05)	**736.91**	(694.99–774.94)
	2015	635.59	(626.46–644.62)	**709.55**	(663.14–751.96)
	2017	627.81	(617.59–637.92)	**698.96**	(662.35–733.20)
	2022	695.27	(689.62–700.85)	**761.75**	(738.09–783.90)
1000 persons living in housing within 8 blocks or 1 km of public transportation	2015	**952.07**	(948.64–955.28)	983.20	(976.58–987.97)
	2017	**939.17**	(934.85–943.22)	977.04	(967.89–983.62)
	2022	**926.29**	(922.85–929.59)	960.47	(951.00–968.17)
1000 persons living in housing within 20 blocks or 2.5 km of a healthcare center	2015	**835.89**	(828.74–842.79)	915.10	(882.94–939.04)
	2017	**840.10**	(831.83–848.04)	917.03	(896.09–934.06)
	2022	**807.45**	(801.79–812.98)	881.37	(864.34–896.52)
1000 persons living in housing within 20 blocks or 2.5 km of an educational institution	2015	**927.96**	(923.23–932.42)	960.03	(929.52–977.65)
	2017	**914.50**	(908.92–919.76)	955.07	(940.50–966.20)
	2022	**907.36**	(903.80–910.81)	948.34	(938.84–956.43)
**Affordability**		*Indicators*		*Indicators*	
Median “Home Value Approximation” Qi / Median Total Household Income Qi (Qi: The quintiles of autonomous household income from 1 to 5, with 1 being the poorest and 5 the richest).	2013	Total: 0.226 (Q1:0.351 Q2:0.27 Q3:0.241 Q4:0.219 Q5:0.185)		**Total: 0.298 (Q1:0.737 Q2:0.561 Q3:0.348 Q4:0.326 Q5:0.233)**	
	2015	Total: 0.232 (Q1:0.363 Q2:0.275 Q3:0.239 Q4:0.224 Q5:0.192)		**Total: 0.319 (Q1:0.838 Q2:0.542 Q3:0.458 Q4:0.352 Q5:0.235)**	
	2017	Total: 0.247 (Q1:0.397 Q2:0.303 Q3:0.266 Q4:0.233 Q5:0.194)		**Total: 0.376 (Q1:1.055 Q2:0.612 Q3:0.537 Q4:0.447 Q5:0.249)**	
	2022	Total: 0.274 (Q1:0.427 Q2:0.322 Q3:0.283 Q4:0.261 Q5:0.222)		**Total: 0.438 (Q1:0.902 Q2:0.627 Q3:0.490 Q4:0.403 Q5:0.293)**	

*Confidence intervals do not overlap between locals and migrants

### Bold lettering: Disadvantaged population in relation to the corresponding housing indicator

After adjusting for sex, age, geographic area, and household autonomous income quintile, being a migrant was associated with a higher chance of living in housing with 2 or more people per bedroom (α = 0.05). The result was consistently worse for the migrant population than for the local population for all included years (Table 3).

A similar situation occurred with regards to living in housing with irrecoverable materiality index, where the chance of reporting living in such housing was significantly higher among migrants in 2015, 2017 and 2022, with ORs between 2.89 and 5.65 tending to increase over the years. For the remaining dimensions, migrants were more likely to have witnessed hazardous situations and to reside in areas close to public transport and healthcare centres, although these differences where not statistically significant in all cases (Table 3).

**Table 3:** OR logistic models for each of the indicators of housing as the dependent variable and migration as the independent variable, adjusted for sex, age, geographical area and income quintile. Chile 2013–2022

	2013		2015		2017		2020		2022	
	OR	CI95%	OR	CI95%	OR	CI95%	OR	CI95%	OR	CI95%
**Availability of services, materials, facilities, and infrastructure**										
Living in housing with sanitation facilities (Ref. without)	0.804	(0.257–2.516)	0.635	(0.218–1,852)	0.755	(0.369–1.548)	0.680	(0.397–1.165)	0.786	(0.565–1.092)
Living in housing with electricity (Ref. without)	0.521	(0.203–1.338)	0.591	(0.209–1.675)	0.640	(0.276–1.480)	-		0.367	(0.213–0.633)**
Living in housing with public water supply (Ref. other supply	0.968	(0.696–1,0.344)	0.374	(0.208–0.672)**	1.160	(0.799–1.682)	0.911	(0.607–1.367)	0.501	(0.361–0.694)**
**Habitability**										
Living in housing with more than 2 people per bedroom (Ref. 2 people or less)	3.329	(2.368–4.680)**	3.956	(3.122–5,012)**	4.674	(3.913–5.583)**	3.619	(3.030–4.323)**	6.135	(5.173–7.277)**
Living in housing with irrecoverable materiality index (Ref. Acceptable o recoverable)	2.014	(0.747–5.434)	2.899	(1.171–7,176)**	4.006	(2.154–7.451)**	-		5.648	(3.527–9.045)**
**Location**										
Having experienced or witnessed alcohol or drug use, drug trafficking, fights or threats on public streets and/or shootings or gunshots at least a few times in the month (Ref. No)	1.053	(0.860–1.289)	1.209	(0.958–1.525)	1.129	(0.954–1.336)	-		1.157	(1.019–1,315)*
Living in housing within 8 blocks or 1 km of public transportation (Ref. No)	-		1.417	(1.031–1,947)*	1.303	(0.941–1.804)	-		1.059	(0.835–1.341)
Living in housing within 20 blocks or 2.5 km of a healthcare center (Ref. No)	-		1.534	(1.015–2.318)*	1.442	(1,134–1.834)**	-		1.412	(1.209–1.649)**
living in housing within 20 blocks or 2.5 km of an educational institution (Ref. No)	-		0.783	(0.363–1.690)	0.994	(0.712–1,389)	-		1.144	(0.928–1.410)

OR model: Housing indicator ~ migration + sex + age + income quintile + area

For all models the F-test (Overall F-fit statistic) indicates that the fitted models are significant, indicating that at least one of the included independent variables is related to the dependent variable

* p-value < 0.05 OR international migration ** p-value < 0.01 OR international migration

Noteworthy, in contrast to the descriptive findings after adjusting for sex, age, geographic area and household income quintile, international migrants were generally less likely to reside in housing with sanitation facilities, electricity, and public water supply, however, only in some years were these differences statistically significant (Table 3).

**Fig. 1:**
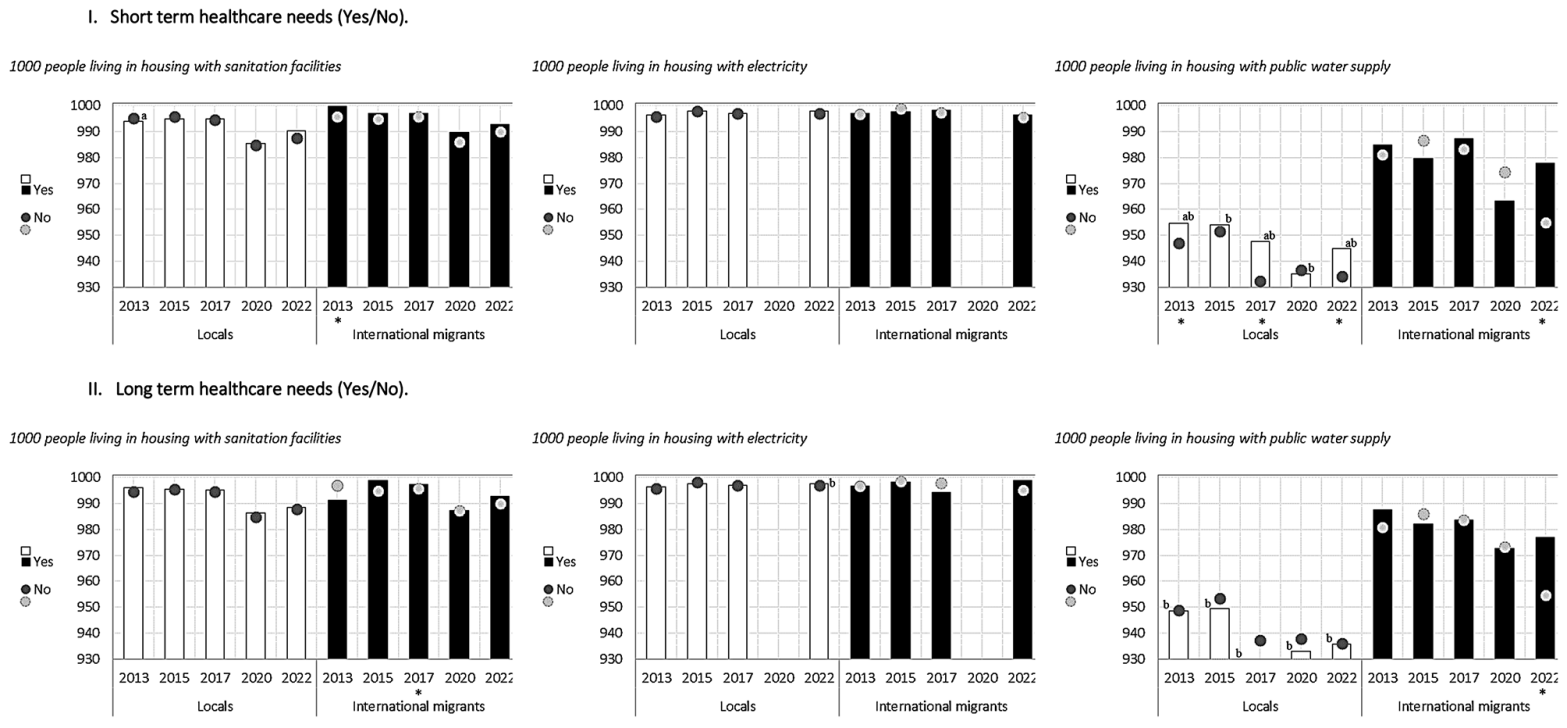
Availability of services, materials, facilities, and infrastructure indicators among local and international migrant populations, distinguishing between those who had short-term healthcare needs and those who did not, and between those who had long-term healthcare needs and those who did not. Chile 2013–2022. * The 95% confidence intervals between those who had a short-term healthcare need and those who did not do not overlap. * Confidence intervals between those who had a long-term healthcare need and those who did not do not overlap. **(a)** The 95% confidence intervals among those with a short-term or long-term healthcare need do not overlap between locals and international migrant populations. **(b)** The 95% confidence intervals among those with no short- or long-term healthcare need do not overlap between local and international migrant populations

**Fig. 2:**
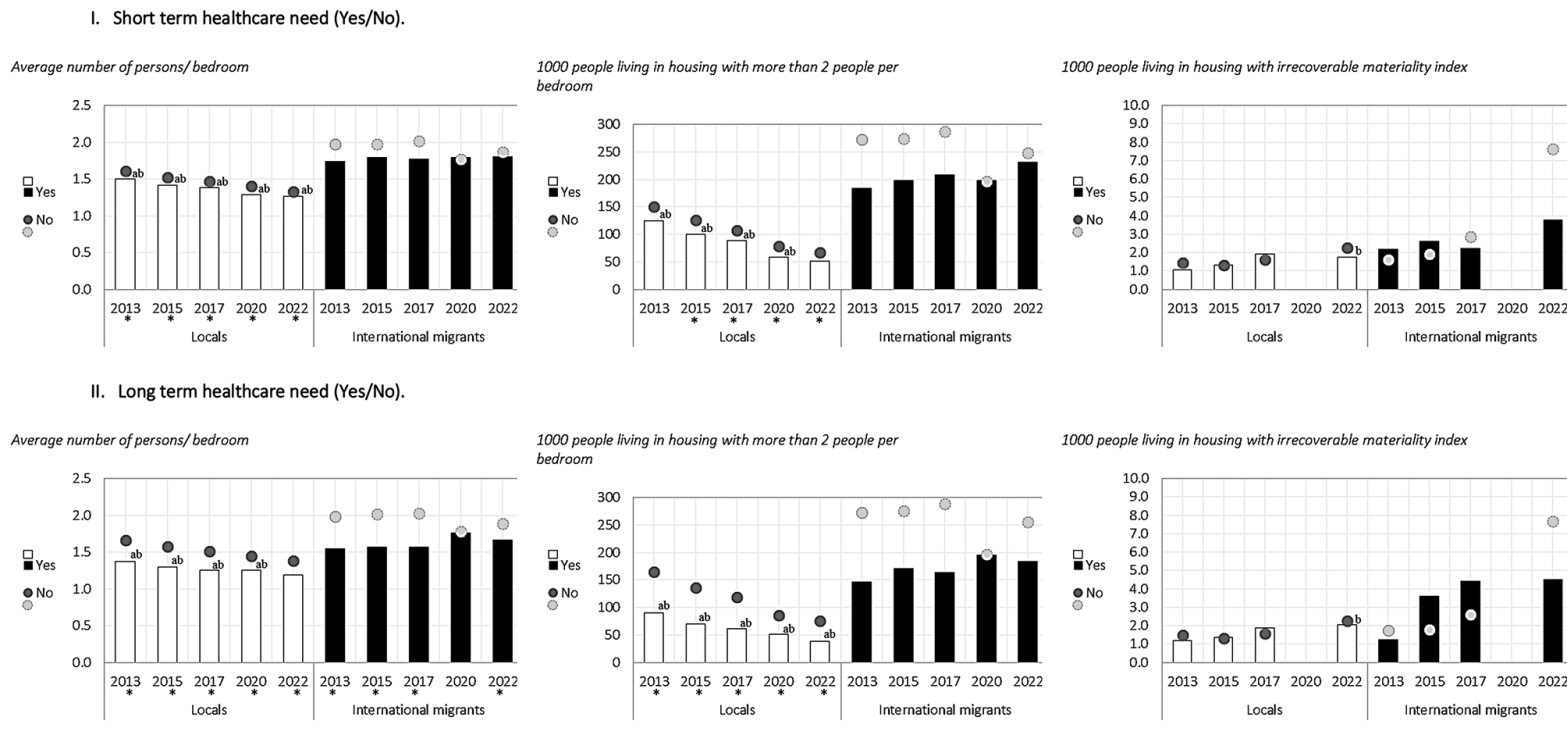
Habitability indicators among local and international migrant populations, distinguishing between those who had short-term healthcare need and those who did not, and between those who had long-term healthcare need and those who did not. Chile 2013–2022. * The 95% confidence intervals between those who had a short-term healthcare need and those who did not do not overlap. * Confidence intervals between those who had a long-term healthcare need and those who did not do not overlap. **(a)** The 95% confidence intervals among those with a short-term or long-term healthcare need do not overlap between locals and international migrant populations. **(b)** The 95% confidence intervals among those with no short- or long-term healthcare need do not overlap between local and international migrant populations

**Fig. 3:**
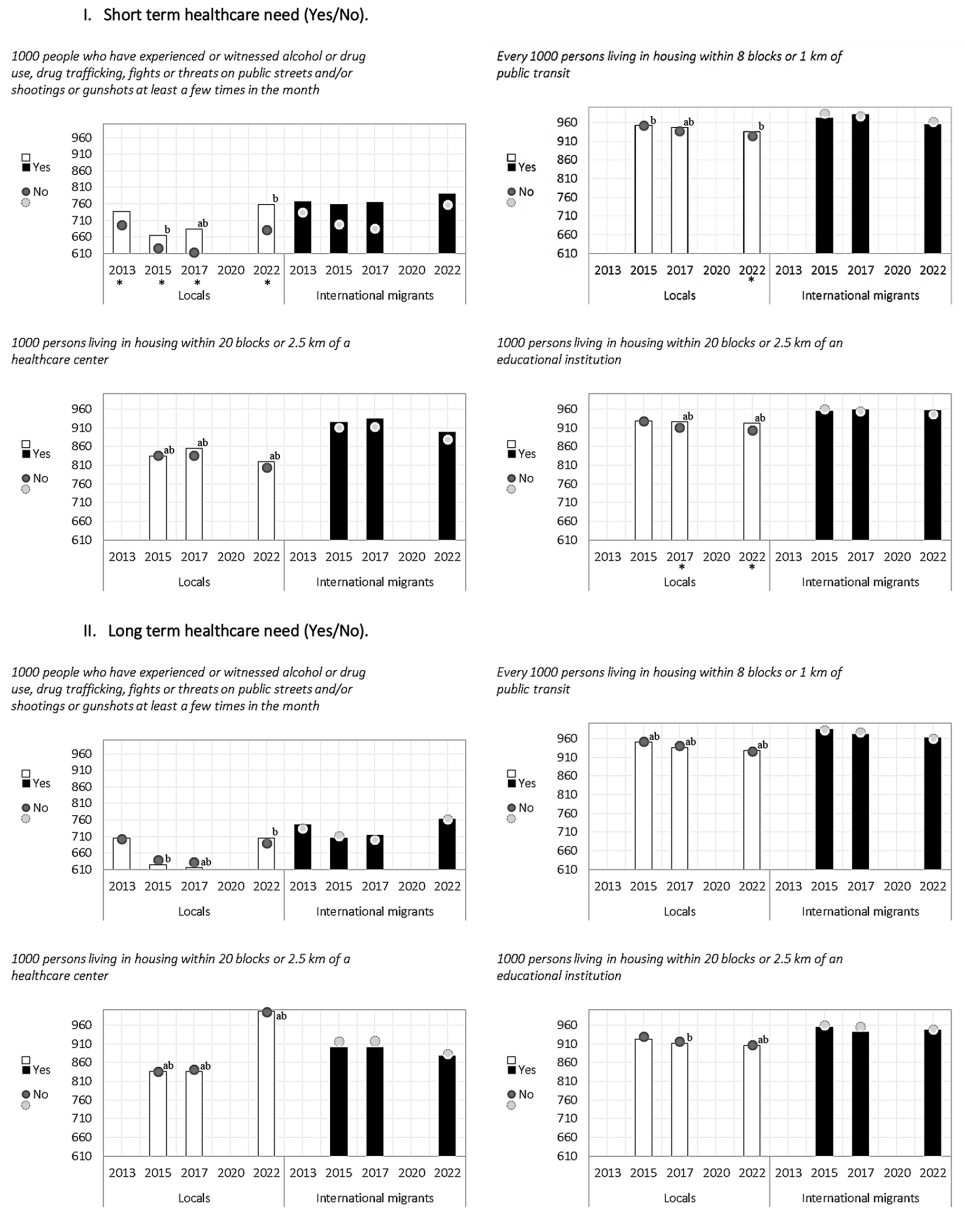
Location indicators among local and international migrant populations, distinguishing between those who had short-term healthcare need and those who did not, and between those who had long-term healthcare need and those who did not. Chile 2013–2022. * The 95% confidence intervals between those who had a short-term healthcare need and those who did not do not overlap. * Confidence intervals between those who had a long-term healthcare need and those who did not do not overlap. **(a)** The 95% confidence intervals among those with a short-term or long-term healthcare need do not overlap between locals and international migrant populations. **(b)** The 95% confidence intervals among those with no short- or long-term healthcare need do not overlap between local and international migrant populations

**Fig. 4:**
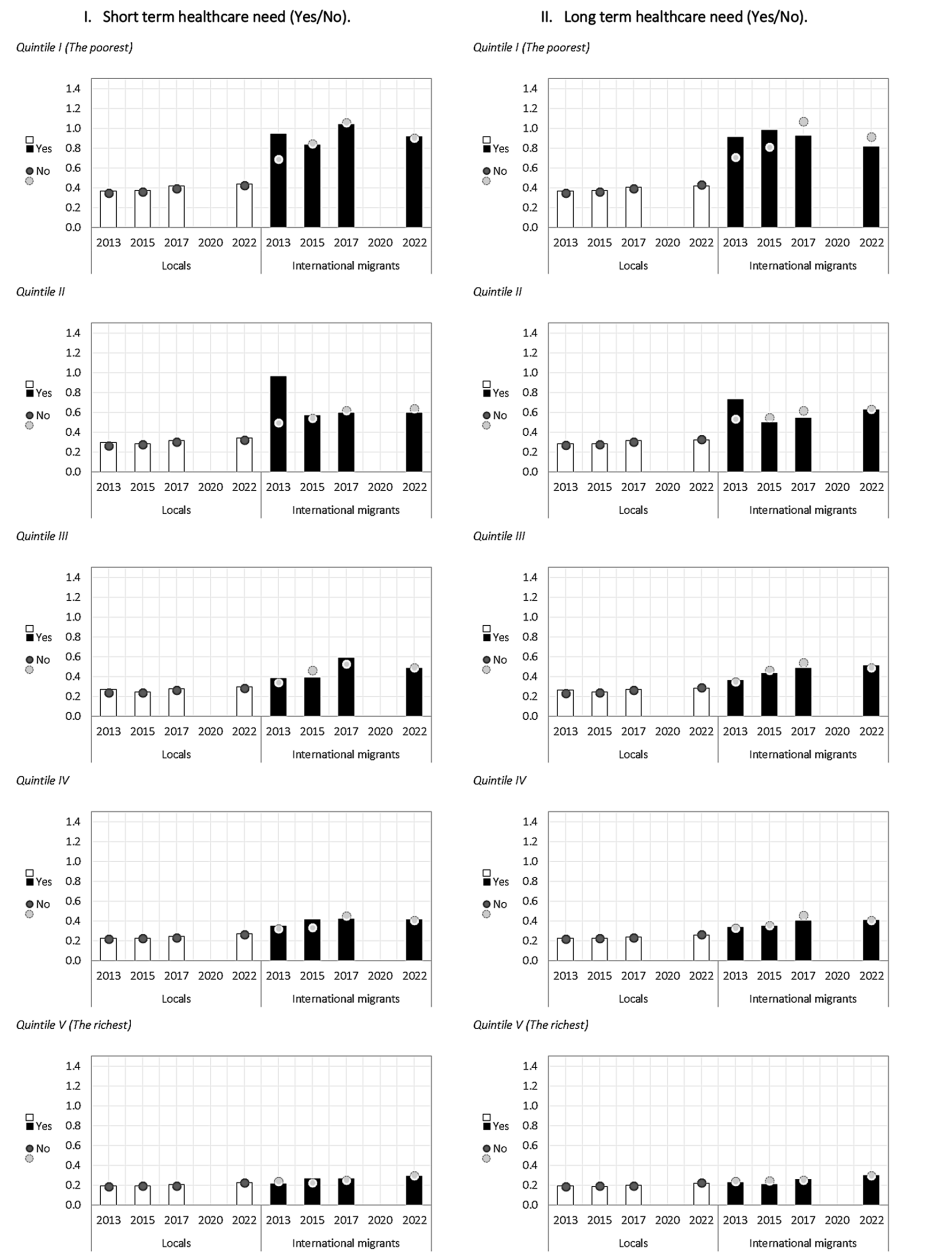
Affordability indicators among local and international migrant populations, distinguishing between those who had short-term healthcare need and those who did not, and between those who had long-term healthcare need and those who did not. Chile 2013–2022

In relation to health outcomes, short-term healthcare need (illness or accident in the 3 months prior to the survey) and long-term healthcare need (receiving treatment for a pathology in the 12 months prior to the survey), some differences were observed according to health need for some years and specific indicators among both the local and migrant population (Figs. 1, 2, 3 and 4).

For short-term healthcare need, the indicator of sanitation facilities showed differences for the international migrant group in 2013; households with public water supply in local population in 2013,2017 and 2022 and migrant population in 2022; number of people per bedroom differed for local population for the years 2013–2022; safety in local population in 2013,2015,2017 and 2022, proximity to public transport in local population in 2022 and proximity to educational institutions in local population in 2017 and 2022 (Figs. 1, 2, 3 and 4).

For long-term healthcare need, possible differences were found in the indicators of sanitation facilities (migrants 2017), public water network (migrants 2022), average number of persons per bedroom (local population 2013 to 2022 and migrant population 201,2015,2017 and 2022) and housing with more than two persons per bedroom (local population 2013 to 2022) (Figs. 1, 2, 3 and 4).

Particularly, for the migrant population, possible differences were found only for the public water network indicator (in 2022 in favour of those with no short-term need and those with no long-term need), in sanitation facilities (in 2013 in favour of those with no short-term need and in 2017 those with no long-term need) and in the average number of persons per bedroom (in 2013, 2015, 2017 and 2022 in favour of those with no long-term need) (Figs. 1, 2, 3 and 4).

In summary, differences in housing indicators varied according to health need, migration status and year of analysis, presenting fluctuant patterns between groups in some cases, with differences found more frequently among the local population than the migrant populations. The figures presented below show, through columns and dots, the housing indicators for locals and international migrants according to whether they reported short- or long-term healthcare needs, so as to observe the behavior of each group in parallel, with respect to a given indicator. Specifically, columns represent the housing indicator for those who reported a healthcare need, and the dots represent those who did not report healthcare needs for the same indicator. The letters (ab) included in the graph determine the significance of the differences between the groups (Figs. 1, 2, 3 and 4).

After adjusting for sex, age, geographic area, income quintile and health provision, only some housing variables per year between 2013 and 2017 were statistically significant for short-term need among international migrants. These variables were living in housing with sanitation facilities in 2013, living in housing close to public transportation in 2015 and witnessing hazardous situations in 2017, all of which implied a risk for reporting short-term healthcare need. In 2020, none of the housing variables remained significant, while in 2022 living in housing with public water supply and having witnessed hazardous situations were significant variables when comparing them between migrants and locals. Regarding the latter, migrants who had witnessed such situations were 1,244 times more likely to reporting a short-term healthcare need than those who had not. In comparison, the local population presented between 1 and 4 significant housing variables per year, highlighting that for all years analysed the number of people per and witnessing hazardous situations were significant in relation to short-term healthcare need. The former was a protective factor and the latter a risk factor (Table 4).

**Table 4:** OR logistic models of short-term healthcare need for each of the housing indicators as an independent variable, adjusted for sex, age, geographic area, household autonomous income quintile and health care provision, in local and international migrant populations. Chile 2013–2022

Short-term healthcare need					
	**2013**	**2015**	**2017**	**2020**	**2022**
Migrant (Ref: Local)	0.770*	0.828*	0.818*	0.825**	0.644**
Local					
**Availability of services, materials, facilities, and infrastructure**					
Living in housing with sanitation facilities (Ref. without)	0.723*	0.761*	0.972	0.992	1.104
Living in housing with electricity (Ref. without)	1.288	1.160	0.967	-	1.151
Living in housing with public water supply (Ref. other supply	1.045	0.987	1.062	0.911	0.900*
**Habitability**					
Number of people per bedroom	0.900**	0.878**	0.952**	0.947*	0.956*
Living in housing with more than 2 people per bedroom (Ref. 2 people or less)	0.859**	0.854**	0.950	0.965	0.915
Living in housing with irrecoverable materiality index (Ref. Acceptable o recoverable)	0.757	1.003	1.291	-	0.933
**Location**					
Having experienced or witnessed alcohol or drug use, drug trafficking, fights or threats on public streets and/or shootings or gunshots at least a few times in the month (Ref. No)	1.231**	1.213**	1.365**	-	1.413**
Living in housing within 8 blocks or 1 km of public transportation (Ref. No)	-	1.024	1.018	-	0.984
Living in housing within 20 blocks or 2.5 km of a healthcare center (Ref. No)	-	0.970	1.025	-	0.966
living in housing within 20 blocks or 2.5 km of an educational institution (Ref. No)	-	1.014	1.058	-	1.061
**Affordability**					
"Home Value Approximation" / Total Household Income	1.000	1.027*	1.009	-	1.003
International migrant					
**Availability of services, materials, facilities, and infrastructure**					
Living in housing with sanitation facilities (Ref. without)	15.000**	1.671	1.028	1.574	1.383
Living in housing with electricity (Ref. without)	1.607	0.576	1.442	-	0.745
Living in housing with public water supply (Ref. other supply	1.399	0.610	0.586	0.727	1.978**
**Habitability**					
Number of people per bedroom	0.820	1.027	0.852	1.097	1.034
Living in housing with more than 2 people per bedroom (Ref. 2 people or less)	0.559	0.922	0.798	1.123	1.076
Living in housing with irrecoverable materiality index (Ref. Acceptable o recoverable)	1.594	1.080	1.583	-	0.621
**Location**					
Having experienced or witnessed alcohol or drug use, drug trafficking, fights or threats on public streets and/or shootings or gunshots at least a few times in the month (Ref. No)	1.245	1.396	1.532*	-	1.244*
Living in housing within 8 blocks or 1 km of public transportation (Ref. No)	-	0.515*	1.139	-	0.752
Living in housing within 20 blocks or 2.5 km of a healthcare center (Ref. No)	-	1.215	1.189	-	1.104
living in housing within 20 blocks or 2.5 km of an educational institution (Ref. No)	-	0.781	0.996	-	0.931
**Affordability**					
"Home Value Approximation" / Total Household Income	1.200	1.196	1.009	-	1.042

OR model: Short-term healthcare need indicator ~ housing variable + gender + age + autonomous income quintile + area + healthcare insurance

For all models the F-test (Overall F-fit statistic) indicates that the fitted models are significant, indicating that at least one of the included independent variables is related to the dependent variable

* p-value < 0.05 OR international migration ** p-value < 0.01 OR international migration

Similarly, for long-term healthcare need among the international migrant population, the significant variables varied across the years, with 2013 having the highest number of significant housing variables (four variables) and 2017 and 2020 having the lowest number (no variable). For both 2013 and 2022 living in housing with public water supply was a risk factor for having long-term healthcare need (OR, 2.84 and 1.71, respectively). Additionally, for 2013 and 20,215 as the number of people per room increased the likelihood of having a long-term health need decreased. The results for long-term healthcare need among locals were again similar to those for short-term healthcare need, whereby there was a higher number of significant housing variables in relation to long-term need. These variables were the number of people per bedroom, having more than two people per bedroom to sleep, and witnessing hazardous situations every year (the first two being protective factors and the second a risk factor) (Table 5).

**Table 5:** OR logistic models of long-term healthcare need for each of the housing indicators as an independent variable, adjusted for sex, age, geographic area, household autonomous income quintile and health care provision, in local and international migrant populations. Chile 2013–2022

Long-term healthcare need					
	2013	2015	2017	2020	2022
Migrant (Ref: local)	0.593**	0.604**	0.495**	0.531**	0.497**
Locals					
**Availability of services, materials, facilities, and infrastructure**					
Living in housing with sanitation facilities (Ref. without)	1.212	0.788	1.166	1.024	1.125
Living in housing with electricity (Ref. without)	1.321	0.993	1.062		1.428**
Living in housing with public water supply (Ref. other supply	1.256**	0.913	1.067	1.001	1.074*
**Habitability**					
Number of people per bedroom	0.891**	0.858**	0.886**	0.878**	0.893**
Living in housing with more than 2 people per bedroom (Ref. 2 people or less)	0.806**	0.798**	0.857**	0.873**	0.826**
Living in housing with irrecoverable materiality index (Ref. Acceptable o recoverable)	0.676	0.716*	0.841		0.788
**Location**					
Having experienced or witnessed alcohol or drug use, drug trafficking, fights or threats on public streets and/or shootings or gunshots at least a few times in the month (Ref. No)	1.223**	1.213**	1.155**		1.206**
Living in housing within 8 blocks or 1 km of public transportation (Ref. No)		1.070	0.983		1.093**
Living in housing within 20 blocks or 2.5 km of a healthcare center (Ref. No)		1.035	1.011		0.976
living in housing within 20 blocks or 2.5 km of an educational institution (Ref. No)		1.071*	1.088*		1.051
**Affordability**					
"Home Value Approximation" / Total Household Income	0.999	1.012	1.005		0.955
International migrants					
**Availability of services, materials, facilities, and infrastructure**					
Living in housing with sanitation facilities (Ref. without)	0.229**	2.539	1.339	0.753	1.218
Living in housing with electricity (Ref. without)	0.748	1.098	0.583		1.239
Living in housing with public water supply (Ref. other supply	2.836**	3.112	1.056	1.041	1.707**
**Habitability**					
Number of people per bedroom	0.762*	0.784**	0.830	1.132	0.958
Living in housing with more than 2 people per bedroom (Ref. 2 people or less)	0.513	0.735	0.862	1.223	1.029
Living in housing with irrecoverable materiality index (Ref. Acceptable o recoverable)	0.916	1.563	1.823		0.540
**Location**					
Having experienced or witnessed alcohol or drug use, drug trafficking, fights or threats on public streets and/or shootings or gunshots at least a few times in the month (Ref. No)	1.097	1.396	1.128		1.154
Living in housing within 8 blocks or 1 km of public transportation (Ref. No)		1.267	1.360		0.854
Living in housing within 20 blocks or 2.5 km of a healthcare center (Ref. No)		0.852	0.950		0.949
living in housing within 20 blocks or 2.5 km of an educational institution (Ref. No)		1.079	0.773		0.836
**Affordability**					
"Home Value Approximation" / Total Household Income	1.213**	0.995	0.866		0.926

OR model: Long-term healthcare need indicator ~ housing variable + gender + age + autonomous income quintile + area + healthcare insuranceFor all models the F-test (Overall F-fit statistic) indicates that the fitted models are significant, indicating that at least one of the included independent variables is related to the dependent variable

* p-value < 0.05 OR international migration ** p-value < 0.01 OR international migration

## Discussion

The present study aimed at analysing adequate housing as a social determinant of health among international migrants in Chile between 2013 and 2022, based on a repeated analysis of CASEN surveys. Adequate housing indicators were selected following those recommended by the United Nations Housing Rights Programme (UNHRP) 2003 report [36], based on the dimensions of adequate housing described by the Committee on Economic, Social and Cultural Rights General Comment No. 4, the Right to Adequate Housing, of 1991. Considering feasibility and availability of data, the following four dimensions of adequate housing were explored: (i) availability of services, materials, facilities, and infrastructure, (ii) habitability, (iii) location, and (iv) affordability. Health was conceptualized as short-term and long-term healthcare need, as having had any illness or accident in the 3 months prior to the survey and being under treatment for any pathology during the year prior to the survey, respectively.

Descriptive results regarding adequate housing showed that generally, the local population reported worse conditions with regards to sanitation, electricity, and public water supply compared to international migrants. Furthermore, Chileans seem to live further away from public transportation, healthcare centres, and educational centres than the international migrant population.

These results may owe to the possibility that a higher proportion of the local population lives in rural areas, while international migrants would tend to live in urban areas with better access to public services as well availability of basic facilities in housing. However, this comes at a price, figuratively and literally. International migrants consistently display worse results for overcrowding and living in materially precarious housing (“irrecoverable materiality index”). They also tend to witness or experience more hazardous situations than the local population, ranging from witnessing alcohol and drug use to gun violence in public areas.

Additionally, these results worsened over the years, whereby overcrowding doubled between 2013 and 2022, the chance of living in housing with irrecoverable materiality index more than doubled during the same period, and the probability of witnessing hazardous situations became statistically significant among international migrants in 2022.

Overcrowding and living in materially precarious housing among international migrants in urban settings has been reported in other studies in Chile and has been explained by dynamics of exclusions linked to migratory status, informal employment, limited support networks, and abusive landlords [37–39]. In turn, these dynamics are fed by, and feed into, the racialization of international migrants in urban areas and their territorial exclusion [34, 37, 40]. Similar situations have been documented in other countries in Latin America, including Argentina and Peru [41–43].

Furthermore, living in materially precarious housing may mean living in old, hazardous buildings, but may also mean living in precariously built, probably informal housing. The results of this study in this respect are especially alarming, as the CASEN survey does not collect data in informal settlements nor among unhoused populations, prisons, hospitals, and shelters [44]. Hence, quality of housing conditions among migrants and local populations found in this analysis might underestimate the reality of those who were consistently excluded from the CASEN survey sampling strategy.

It is important to note that inequities become especially visible when it comes to affordability, especially for the poorest quintile among international migrants, as the average value of their housing tend to represent a higher proportion of their average income (over 90% for migrants Q1 vs. 42% for locals Q1 in 2022 for instance). This particular inequity has also been documented in other studies in Chile and it has been suggested that it is one of the reasons for the revival of informal settlements in the past few years [32]. Additionally, this suggests that when disaggregated by quintiles, inequities between Chileans and international migrants may be bigger among the poorest quintiles.

With regards to healthcare needs, overcrowding and experiencing hazardous situations was statistically significant for the local population across all years, and among international migrants, the latter became statistically significant for short-term healthcare needs in 2022. However, in relation to other dimensions of adequate housing, results for the migrant population did not follow a clear pattern, even though descriptive results showed that they tended to live in worse conditions. This may have at least three plausible explanations. First, as the CASEN survey relies on self-reporting, there might be some level of under-reported needs. Second, sample sizes are smaller for the migrant population than for their local counterparts affecting the statistical power of some of the observed associations. Third, an arguable “healthy immigrant effect” may also be at play, where international migrants are usually younger and in better health, at least initially, than the local population [45]. This may be the case with regards to age, as there is a higher representation of population between 15 and 44 years old among the migrant population than the locals in 2015 and 2017 [46, 47]. As the results are not stratified by age, country of origin, time in Chile, and migratory status, it is difficult to discern whether it is the case. Future studies could expand these analyses by considering those variables when available.

The results of this study show international migrants, although a highly heterogeneous population group, tend to experience worse living conditions than their local peers, especially among lowest income quintiles. Furthermore, living in urban areas, where services and employment are concentrated, usually means greater exposure to hazardous situations, with repercussions on their healthcare needs. Examining housing as a social determinant of health among international migrants shows us that some inequities are exacerbated compared with the local population, potentially due to factors linked to the migratory process: migratory irregularity, informal employment, limited support networks, and the risk of experiencing xenophobia as well as rights violation. These factors are, in turn, connected to systemic dynamics of racial exclusion based on the colonial legacy of Chile and other Latin American countries, and of socioeconomic exclusion as a remnant of historical privatisation processes that have taken place in the past decades. Improving housing inequities thus requires parallel processes of profound systemic change to deconstruct oppressive structures, as well as progressive, intersectoral work to eliminate the disadvantages international migrants may face, through the removal or lowering of the barriers for migratory regularisation and for access to essential services, including affordable adequate housing.

### Strengths and limitations

To the best of our knowledge, this is the first study carried out in Chile based on a repeated analysis of CASEN surveys between 2013 and 2022 aiming at analysing housing as a social determinant of health for international migrant populations. The results shed light on persistent and sometimes worsening inequities with regards to adequate housing as part of the right to adequate standards of living between local and international migrants and Chile and highlight affordability as a particularly relevant dimension of adequate housing as a social determinant of health among international migrants. However, some of the results may be nuanced by several limitations. First, the fact that the CASEN surveys do not include informal settlers nor unhoused people may mean that the reality regarding availability of services, materials, facilities, and infrastructures as well as habitability may be worse than shown by the results presented here, with potential effects on healthcare needs. Second, this study did not disaggregate by age, not by more specific variables linked to migration such as time in Chile and migratory status, potentially hiding a “healthy immigrant effect”. Third, due to availability of data in the CASEN database, some dimensions of adequate housing as described by the Committee, such as cultural relevance, were not explored in the current study, meaning that other factors may come into play. Finally, it is worth mentioning that due to its design, the study cannot establish causality, only associations.

Nevertheless, this study represents a novel and unique analysis of housing as a social determinant of health among international migrants compared to locals in Chile and presents original results that are relevant to researchers in the field as well as policymakers at the national level and in Latin America.

## Conclusions

In the historically challenging and contentious context of (in)adequate housing in Latin America, examining adequate housing in relation to health among international migrants in Chile requires considering the ways in which migratory processes may compound disadvantages with regards to the local population. Although international migrants may display certain advantages with regards to access to services, it is largely mitigated by inequities in affordability indicators, overcrowding, materiality, and witnessing hazardous situations, all of which have been worsening over the years. Addressing housing as a social determinant of health among international migrants in Chile requires addressing inequities stemming from the (mis)management of migratory flows and the growing restrictions on migratory regularization, which drive international migrants to face increasingly precarious housing.

## Data Availability

The data is freely available through the following link: https://observatorio.ministeriodesarrollosocial.gob.cl.
